# Systematic tracking of altered haematopoiesis during sporozoite-mediated malaria development reveals multiple response points

**DOI:** 10.1098/rsob.160038

**Published:** 2016-06-22

**Authors:** Maria L. Vainieri, Andrew M. Blagborough, Adam L. MacLean, Myriam L. R. Haltalli, Nicola Ruivo, Helen A. Fletcher, Michael P. H. Stumpf, Robert E. Sinden, Cristina Lo Celso

**Affiliations:** 1 Department of Life Sciences, Imperial College London, South Kensington Campus, London SW7 2AZ, UK; 2 Jenner Institute, Oxford OX3 7DQ, UK

**Keywords:** haematopoietic stem cells, population dynamics, plasmodium infection

## Abstract

Haematopoiesis is the complex developmental process that maintains the turnover of all blood cell lineages. It critically depends on the correct functioning of rare, quiescent haematopoietic stem cells (HSCs) and more numerous, HSC-derived, highly proliferative and differentiating haematopoietic progenitor cells (HPCs). Infection is known to affect HSCs, with severe and chronic inflammatory stimuli leading to stem cell pool depletion, while acute, non-lethal infections exert transient and even potentiating effects. Both whether this paradigm applies to all infections and whether the HSC response is the dominant driver of the changes observed during stressed haematopoiesis remain open questions. We use a mouse model of malaria, based on natural, sporozoite-driven *Plasmodium berghei* infection, as an experimental platform to gain a global view of haematopoietic perturbations during infection progression. We observe coordinated responses by the most primitive HSCs and multiple HPCs, some starting before blood parasitaemia is detected. We show that, despite highly variable inter-host responses, primitive HSCs become highly proliferative, but mathematical modelling suggests that this alone is not sufficient to significantly impact the whole haematopoietic cascade. We observe that the dramatic expansion of Sca-1^+^ progenitors results from combined proliferation of direct HSC progeny and phenotypic changes in downstream populations. We observe that the simultaneous perturbation of HSC/HPC population dynamics is coupled with early signs of anaemia onset. Our data uncover a complex relationship between *Plasmodium* and its host's haematopoiesis and raise the question whether the variable responses observed may affect the outcome of the infection itself and its long-term consequences on the host.

## 1. Introduction

Haematopoietic stem cells (HSCs) maintain the production of red blood cells (RBCs), white blood cells (WBCs) and platelets throughout the lifespan of vertebrates. Highly quiescent, long-term (LT) repopulating stem cells are at the apex of the haematopoietic developmental cascade. However, their downstream progeny, short-term (ST) repopulating stem cells and progenitor cell populations, become progressively more proliferative [1]. Progressive lineage commitment is acquired by progenitor cells and eventually all haematopoietic cell lineages are specified and mature within the bone marrow, from where fully differentiated cells are released into the circulation at a rate of several billion cells/day [2]. The dynamics of this complex hierarchy of cell lineages change during normal development (ageing) and in response to infection by pathogens.

Infections and inflammatory cytokines act directly on HSCs, causing them to proliferate and boost haematopoietic cell production at the same time as immune cells are recruited into the peripheral circulation by immune responses [3,4]. However, this is most often detrimental to HSCs. During severe/chronic infection, LPS, TNFα, interferon (IFN)α and IFNγ and TLR signalling have been linked to HSC proliferation and loss of HSC function, assessed as the ability to engraft in lethally irradiated recipient mice [5–9]. This is usually accompanied by a marked, and not yet completely understood, increase in the Lin^–^c-Kit^+^Sca-1^+^ cell population, together with anomalies in the committed progenitor cell populations, ranging from dramatic loss of myeloid progenitors [6–10] to emergence of atypical myeloid-primed, lymphoid-like progenitors [11]. In some cases, this is accompanied by the loss of myeloid cells from the bone marrow [8], reduced neutrophil production [8] or imbalanced myelopoiesis [12].

By contrast, acute, non-lethal infections have been reported to induce only transient perturbations of haematopoiesis [10]. Acute pneumovirus infection induces intermediate levels of Sca-1 upregulation and has no effect on bone marrow engraftment [13], and we reported increased LT engraftment of haematopoietic bone marrow cells harvested from mice infected by the parasite *Trichinella spiralis* [14]. Increased myeloid production was shown to be mediated by IFNγ during acute infection by the bacterium *Ehrlichia muris* [10] and by TNFα and IFNγ during pneumovirus infection [13].

Major questions still requiring resolution include whether diverse infections perturb haematopoiesis similarly, how the HSC response contributes to stressed haematopoiesis during infection, and whether simultaneous responses across multiple levels of the haematopoietic tree cooperate to support the immune response to pathogens. Here, we used a mouse pathogen, *Plasmodium berghei*, as our experimental model, because its effect on HSCs had not yet been studied, and it might provide information relevant for human malaria, still a widespread disease. Malaria is a severe and life-threatening infection, which can result in cerebral complications and/or anaemia. It is initiated by bites from infected mosquitoes, which inoculate sporozoite parasites in the mammalian host's skin. Sporozoites migrate to the liver, where each parasite generates approximately 15 000 daughter parasites (merozoites) within 45 h. Merozoites invade and disrupt RBCs, causing anaemia [15], while the immune response mounted against the parasites is the cause of cerebral complications due to clogging of blood vessels in the brain [16–18]. Experimentally, malaria can be induced in rodents either by the natural route of mosquito bites, or by direct injection of infected blood.

Early studies on haematopoiesis following inoculation of infected RBCs (iRBCs) showed that malaria infection induces changes in multiple blood cell populations [19–23], but also in earlier haematopoietic cell populations, suggesting that anaemia may partly result from a systemic disruption of haematopoiesis [15,24]. Extensive immunophenotypic characterization of haematopoietic stem and progenitor cells allowed in recent years identification of an anomalous population of IL7Rα^+^c-Kit^Hi^ myeloid-primed progenitors that contributed to the clearance of iRBCs during *Plasmodium chabaudi* infection [11]. However, little is known about the dynamics of the most primitive HSCs in response to *Plasmodium* infection.

All these studies miss the liver stage of disease and are based on an injection that transfers not just parasites but also cellular and humoral components of the immune system of the previously infected donor animal, which may proffer immediate and unnatural responses in congenic hosts [25,26]. To understand the early haematopoietic responses to *Plasmodium* infection and how they develop throughout the natural course of disease progression, we therefore have chosen to use the natural route of sporozoite inoculation in C57/B6 mice by the bite of mosquitoes infected with *P. berghei*. We have examined the changes induced upon haematopoietic stem and progenitor cell populations in the bone marrow, and relate these to the downstream impact upon the RBC and WBC populations in the peripheral blood, spleen and bone marrow. We find that multiple components of the haematopoietic tree simultaneously respond to/are affected by the infection, with HSCs and early progenitors dramatically increasing their proliferative state, more committed myeloid populations being lost and a pre-anaemic stage developing in the bone marrow.

## 2. Material and methods

### 2.1. Parasite maintenance

Routine parasite maintenance within mice was carried out as previously described [27]. *Plasmodium berghei* ANKA 2.34 was maintained in 4–10 week old female Tuck CD1 mice (Charles River) by serial blood passage (up to a maximum of eight passages) and according to Home Office approved protocols. Hyper-reticulocytosis was induced 2–3 days before infection by treating mice with 200 µl i.p. phenylhydrazine chloride (PHz; 6 mg ml^−1^ in PBS; ProLabo, UK). Stock mice were infected by i.p. injection of blood containing parasites, and infections were monitored on Giemsa-stained tail blood smears as described previously [27].

### 2.2. Generation of infected mosquitoes and sporozoite-derived infection of mice

For each individual experiment, a group of five 4–10-week-old female PHz-treated CD1 mice were infected with *P. berghei* ANKA 2.34 by syringe inoculation (i.p.), followed by feeding to mosquitoes at day 3 post-infection. On day 3, five infected mice were anaesthetized and exposed to cages containing 500 starved female *Anopheles stephensi* (SD 500) mosquitoes. Unfed mosquitoes were removed and fed ones were maintained on 8% (w/v) fructose, 0.05% (w/v) p-aminobenzoic acid at 19°C and 80% relative humidity. Mosquitoes were maintained until 21 days post-infection, when salivary gland sporozoites were at their peak [27]. To infect mice with *P. berghei*, individual anesthetized naive C57/B6 mice (Harlan) were exposed to five potentially infected mosquitoes, selected at random from the larger population. Successful feeding was confirmed by the presence of blood in the abdomen of mosquitoes after 20 min. Post-feeding, individual mosquitoes were dissected to determine the presence of salivary gland sporozoites. Mice were deemed infected if they received five potentially infectious bites (confirmed by the presence of salivary gland sporozoites), whereas control mice received five mosquito bites from naive, non-infected mosquitoes in parallel.

Noting that RBCs in infected mice are potentially invaded (at the earliest) at 45 h after infection from a *P. berghei*-infected bite (post-liver stage) [28], we anticipate that properties induced by blood infection will be delayed by approximately 2 days when compared with infections raised by direct blood inoculation as described in previous studies [11,20,22].

### 2.3. Peripheral blood analysis

Parasitaemia was monitored at days 0, 3, 7, 10 post-infection by Giemsa-stained thin blood smears and is expressed as a percentage of more than 500 RBCs counted per slide. Reticulocytes were visualized by staining on acridine orange-coated slides [29]. RBC and WBC counts were determined by diluting 20 µl blood samples from cardiac puncture in heparin and counting them in a haematology analyser (Sysmex X-100) or ADVIA 2120 haematology analyser (Siemens Diagnostics).

### 2.4. Flow cytometry

For fluorescence-activated cell sorting analysis, bone marrow cells were harvested from femurs and tibias of control and infected mice, and resuspended in PBS supplemented with 2% FBS (Gibco) at a cell density of 25 × 10^6^ ml^−1^. Single-cell suspensions were stained with the monoclonal antibodies listed in the electronic supplementary material, table S1 as previously described [7,30]. For RBC development analysis, dead cells were eliminated from the analyses by counterstaining with DAPI (Life Technologies). Apoptotic cells were detected by including Annexin V PE (BD Biosciences) and 7-amino-actinomycin D (7AAD, BD Biosciences) according to the manufacturer's instructions.

For proliferation analysis, infected and control mice were administered 1 mg 5-bromodeoxy-uridine (BrdU, Sigma) per 6 g of body weight i.p. 12 h prior to analysis [9]. BrdU staining was obtained using the BrdU-APC kit (BD Biosciences) following the manufacturer's instructions. A BD LSRFortessa analyser was used to collect all flow cytometry data, and analysis was performed with FlowJo software (Tree Star).

### 2.5. Statistical analysis

Data are expressed as means ± s.e.m. In order to account for variations in cell numbers due to differences in the type and number of bones harvested for different experiments and to show our entire dataset, for some stainings cell numbers were normalized by dividing each value by the average of the corresponding control values for that day. Control stage I values were used to normalize RBC staining data.

Two tailed, unpaired *t*-test was used to analyse data with unequivocal normal distribution, i.e. parasitaemia and spleen weight. For all other data, because not all datasets had normal distribution, the Kolmogorov–Smirnov nonparametric test was used for statistical comparison between uninfected and malaria-infected mice each day. *p*-Values less than 0.05 were considered statistically significant.

### 2.6. Statistical and mathematical modelling

In order to estimate the increase within a cell population due to proliferation *alone* during the time interval [*t*,*t* + 1], we use
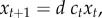
where *x* is the population size, *d* is the cell cycle duration and *c_t_* is the cycling fraction of cells (BrdU+) at time *t*. Note that *x_t_*_+1_ does not necessarily correspond to the total population size at time *t* + 1, as it does not take into account other processes including differentiation and cell death.

We model the dynamics of haematopoiesis in a highly idealized manner in which four distinct cell populations interact, namely HSCs (*S*), multipotent and committed progenitor cells (*P*), and red (*R*) and white (*W*) blood cells. The ordinary differential equations that specify this model are:
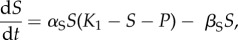

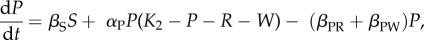

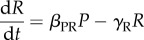

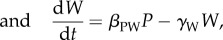
where *α*_S_, *β*_S_, *α*_P_, *β*_PR_, *β*_PW_, *γ*_R_, *γ*_W_, *K*_1_ and *K*_2_ are parameters of the model. *K*_1_ and *K*_2_ are carrying capacities used to define the compartments containing stem and progenitor cells and progenitor and differentiated cells, respectively. For simulations, we use *K*_1_ = 10^3^ and *K*_2_ = 10^5^. The remaining parameters were set such that expected population sizes were recovered (where the total population sizes have been scaled by a factor of 10^−3^): *S* ≈ 10, *P* ≈ 10^3^, *R* ≈ 10^5^, *W* ≈ 10^4^.

## 3. Results

### 3.1. Haematopoietic stem and progenitor cells numbers are affected by sporozoite-mediated *Plasmodium berghei* infection

**Figure 1.**
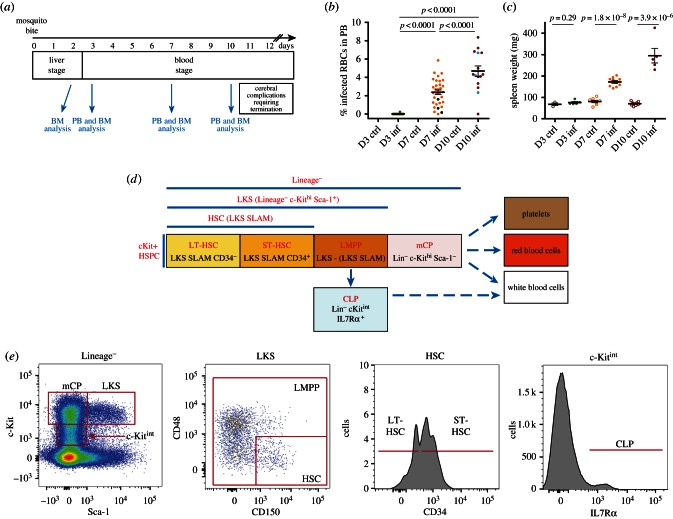
Analysis of the haematopoietic response to sporozoite *P. berghei* infection. (*a*) Timeline of *P. berghei*-induced malaria onset and time point analysed. On day 0, cohorts of C57/B6 mice were exposed to bites by control or *P. berghei*-infected *A. stephensi* mosquitoes. On days 3, 7 and 10 psi, groups of 2–3 control and 3–5 infected mice were culled and their peripheral blood (PB) and bone marrow (BM) cells analysed. Boxes indicate the duration of liver/blood stages of disease and the time of onset of cerebral complications. In this study, we analysed animals from a total of six independent infections. (*b*) Parasitaemia detected at days 3, 7 and 10 psi. *p*-Values are not shown but all <0.005 for each pairwise comparison. Black dots in the day 7 pool and light blue dots in the day 10 pool indicate mice that, despite showing parasitaemia, did not mount a dramatic haematopoietic response. *n* = 20 mice culled and analysed at day 3, 32 at day 7 and 15 at day 10 psi, pooled from six independent infections. (*c*) Spleen weight for control and infected mice at the times indicated. *n* = 5 mice culled and analysed at day 3, 10 at day 7 and 5 at day 10 psi, pooled from three independent infections. (*d*) Schematic of the haematopoietic stem and progenitor cell populations analysed, including phenotypic markers and nomenclature used throughout the manuscript. HSC, haematopoietic stem cells (LT, long-term, ST, short-term repopulating); LMPP, lymphoid and myeloid multipotent progenitors; mCP, myeloid committed progenitors; CLP, common lymphoid progenitors; LKS, Lineage^−^Kit^hi^Sca^+^; SLAM: CD150^+^CD48^−^. (*e*) Gates used to identify the cell populations in (*d*) using flow cytometry analysis. Above each plot is indicated the population shown, and boxes indicate how subpopulations were identified based on the expression levels of cell surface markers.

To gain a comprehensive view of the responses to *P. berghei* infection in haematopoietic stem and progenitor cell populations, we performed flow cytometry analysis of multiple bone marrow haematopoietic populations at days 2, 3, 7 and 10 post-sporozoite infection (psi). These time points were selected to look for perturbations taking place during the liver stage of disease (day 2), at times when parasitaemia is barely and then clearly detectable (day 3 and 7, respectively), and when the immune response is strongly engaged (day 10), but before escalating into cerebral malaria [31] (figure 1*a*). As expected, parasitaemia (i.e. the percentage of infected erythrocytes in the blood) was rarely detectable on day 3, but increased by day 7 and 10 psi to averages of 2.4% (±0.25 s.e.m.) and 4.7% (±0.56 s.e.m.), respectively (figure 1*b*). Two animals showing no parasitaemia, one at day 7, the other at day 10, were excluded from further analysis as they were deemed uninfected. As noted in earlier studies, splenomegaly was invariably observed following infection. Spleen size (data not shown) and weight (figure 1*c*) doubled by day 7 and nearly quadrupled by day 10 psi.

We used the LKS (Lineage^−^c-Kit^+^Sca-1^+^) SLAM marker (CD48^−^CD150^+^) combination to identify HSCs as it has been demonstrated that SLAM markers expression is not affected by stress [7,9,32], and CD34 expression to separate LT- and ST-HSCs [1,7,33,34]. HSC progeny, still falling within the LKS phenotype (but outside the SLAM-defined HSC population) have reduced self-renewal potential, but give rise to both myeloid and lymphoid cells. We therefore refer to them as lymphoid/myeloid primed multipotent progenitors (LMPPs). Further downstream progeny of LMPPs include committed myeloid and lymphoid progenitors, which during steady-state haematopoiesis express lower levels of the cell surface marker Sca-1 [35]. All committed myeloid progenitors together can be identified as Lin^−^c-Kit^+^Sca^−^ (we refer to this overall population as mCP for myeloid committed progenitors) [35]. We use the term ‘c-Kit^+^ HSPCs’ to refer to the whole, mixed population of HSCs, LMPPs and mCPs (figure 1*d*,*e*).

**Figure 2.**
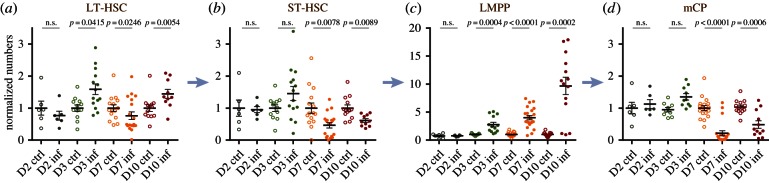
Changes in HSPC populations during malaria onset. Each dot plot shows data collected from each control/infected mouse analysed at the days psi indicated. Arrows indicate the hierarchical relationship between the cell populations analysed. (*a*) LT-HSC numbers, *n* = 4, 12, 15, 13 control and 6, 14, 19, 10 infected mice culled and analysed at days 2, 3, 7 and 10 psi, respectively, pooled from six independent infections. (*b*) ST-HSC numbers, *n* = 4, 12, 15, 13 control and 6, 14, 19, 10 infected mice culled and analysed at days 2, 3, 7 and 10 psi respectively, pooled from six independent infections. (*c*) LMPP numbers, *n* = 4, 11, 21, 17 control and 6, 13, 22, 14 infected mice culled and analysed at days 2, 3, 7 and 10 psi, respectively, pooled from seven independent infections. (*d*) mCP numbers, *n* = 4, 12, 21, 17 control and 6, 13, 23, 14 infected mice culled and analysed at days 2, 3, 7 and 10 psi, respectively, pooled from seven independent infections. *p*-Values for comparison between infected and control values each day are as indicated. n.s., not significant (*p* > 0.05).

We observed that the number of LT-HSCs fluctuated during the course of infection: a small but significant elevation in the LT-HSC compartment was detected at day 3 psi, reverted to a slight decline at day 7 psi (*p* = 0.0246), and reappeared significantly at day 10 psi (figure 2*a*). The ST-HSC population, in contrast, was significantly reduced relative to controls as the infection progressed (day 7 and 10 psi; figure 2*b*). As a result, the overall HSC population (LKS SLAM) decreased by about 50% at day 7 psi, but had returned to normal by day 10 psi (electronic supplementary material, figure S1). LMPPs showed the most consistent and robust response to infection, with a steady increase up to a 10-fold average by day 10 psi (figure 2*c*). The corresponding increase in the global LKS population (electronic supplementary material, figure S1*a*) is consistent with previous observations made with severe infections or high doses of inflammatory cytokines [3,4]. An opposing trend was observed for mCPs, which dropped dramatically at day 7 psi, followed by a partial recovery by day 10 psi (figure 2*d*). Of note, 3/23 mice at day 7 and 3/14 at day 10 psi did not show raised LMPP/LKS populations nor loss of mCPs, nor a reduction in the proportion of HSCs within the LKS gate (figure 2 and electronic supplementary material, figure S1). This was due to a lack of Sca-1 upregulation despite parasitaemia being similar to other mice (see black and light blue dots in figure 1*b*).

Taken together, these data suggest that *P. berghei* infection dramatically affects multiple stages of the haematopoietic hierarchy simultaneously, and from very early stages of infection. Moreover, they raise the questions of what cellular dynamics could be responsible for the changes observed in the stem and progenitor cell compartments, and whether the increase in LKS population and loss of mCPs could be linked.

### 3.2. Proliferation of distinct HSPC populations differentially increases following *Plasmodium berghei* infection

**Figure 3.**
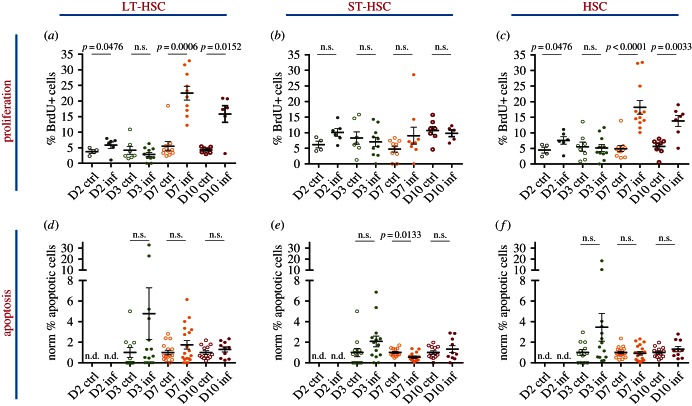
HSC and HSC subpopulations: proliferation and apoptosis during sporozoite *P. berghei* infection. (*a*–*c*) Percentage of BrdU+ cells in LT-HSC (*a*), ST-HSC (*b*) and the overall HSC (*c*) populations analysed in control and infected mice at days 2, 3, 7 and 10 psi. *n* = 4, 7, 10, 7 control and 6, 10, 10, 6 infected mice analysed at days 2, 3, 7 and 10 psi, respectively, in (*a*); 4, 7, 10, 7 control and 6, 10, 9, 5 infected mice analysed at days 2, 3, 7 and 10 psi, respectively, in (*b*); and 11, 14, 10 control and 14, 15, 10 infected mice analysed at days 3, 7 and 10 psi, respectively, in (*c*). Data are pooled from three to six independent infections. (*d*–*f*) percentage of AnnexinV^+^7AAD^−^ apoptotic cells in LT-HSC (*d*), ST-HSC (*e*) and the overall HSC (*f*) populations analysed in control and infected mice at days 3, 7 and 10 psi. *n* = 11, 16, 13 control and 15, 19, 10 infected mice analysed at days 3, 7 and 10 psi, respectively, in (*d*); 12, 13, 13 control and 14, 15, 10 infected mice analysed at days 3, 7 and 10 psi, respectively, in (*e*) and 12, 18, 13 control and 14, 19, 10 infected mice analysed at days 3, 7 and 10 psi, respectively, in (*f*). Data are pooled from three to five independent infections. *p*-Values for comparison between infected and control values each day are as indicated. n.s., not significant (*p* > 0.05).

To shed light on the potential mechanisms behind the dynamics of HSPC cell population changes in response to *P. berghei* infection, we queried the same populations for their proliferation and apoptosis. Infected and control mice were administered BrdU 12 h prior to bone marrow harvest and the proportion of BrdU+ cells in each compartment was measured to obtain a qualitative indication of the amount of proliferation. Despite a small but statistically significant increase in the proportion of BrdU+ LT-HSCs at day 2 psi, no changes were detected in either the LT- or ST-HSCs at day 3 psi; however, a significant proportion of LT-HSCs incorporated BrdU at later time points (approx. 20% at day 7 psi and 15% at day 10 psi; figure 3*a*). By contrast, the proportion of BrdU+ ST-HSCs remained unchanged throughout our analysis (figure 3*b*), thus the combined HSC population exhibited 3.5- and 2.5-fold increases in BrdU+ cells at days 7 and 10 psi, respectively (figure 3*c*). Despite the elevation in BrdU incorporation, the proportion of live, AnnexinV^+^ LT-, ST- and all HSCs appeared unchanged throughout our analysis. While recognizing the limitations of an *ex vivo* analysis of apoptosis, these data may suggest that apoptosis does not play a significant role in driving the observed changes in HSC numbers (figure 3*d*–*f*).

**Figure 4.**
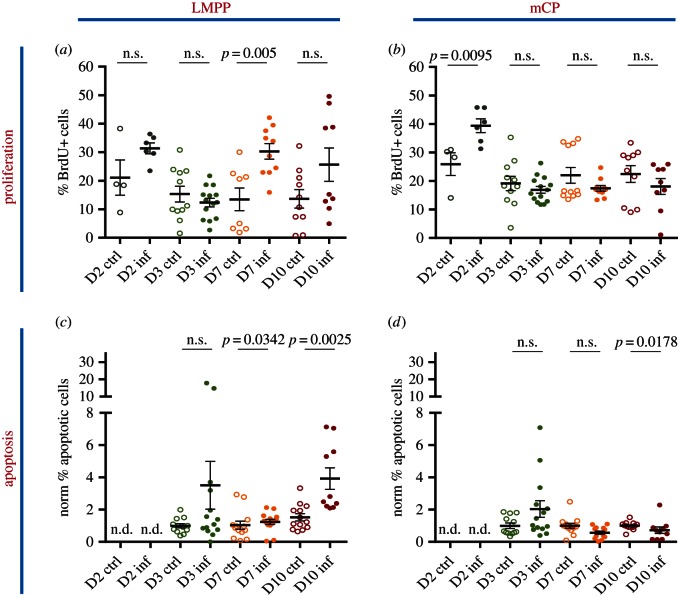
Proliferation and apoptosis of multipotent and myeloid committed haematopoietic progenitor populations in response to sporozoite *P. berghei* infection. (*a,b*) Percentage of BrdU + LMPP (*a*) and mCP (*b*) populations in control and infected mice analysed at days 2, 3, 7 and 10 psi. *n* = 4, 11, 8, 9 control and 6, 14, 10, 9 infected mice analysed at days 2, 3, 7 and 10 psi, respectively, in (*a*); 4, 11, 11, 9 control and 6, 14, 10, 9 infected mice analysed at days 2, 3, 7 and 10 psi, respectively, in (*b*). Data pooled from five independent infections. (*c,d*) Percentage of percentage of AnnexinV^+^7AAD^−^ apoptotic cells in LMPP (*c*) and mCP (*d*) populations. *n* = 12, 13, 13 control and 14, 14, 10 infected mice analysed at days 3, 7 and 10 psi, respectively. Data pooled from four independent infections. *p*-Values for comparison between infected and control values each day are as indicated. n.s., not significant (*p* > 0.05).

Upon examination of the more differentiated progenitors, we observed that a significant portion of the LMPP compartment incorporated BrdU at day 7 psi. By day 10, an interesting bi-modal distribution was evident, such that the LMPP population was highly proliferative in some mice, and relatively non-dividing in others (figure 4*a*). Notably, all four highly proliferative mice harboured an increased proportion of apoptotic, AnnexinV^+^ LMPPs at this time point (figure 4*c*). The proportion of proliferative mCPs rose on day 2 psi but the number of apoptotic cells decreased at day 10 psi (figure 4*b*,*d*).

### 3.3. *Plasmodium berghei* infection leads to mixing of LMPP and mCP populations

**Figure 5.**
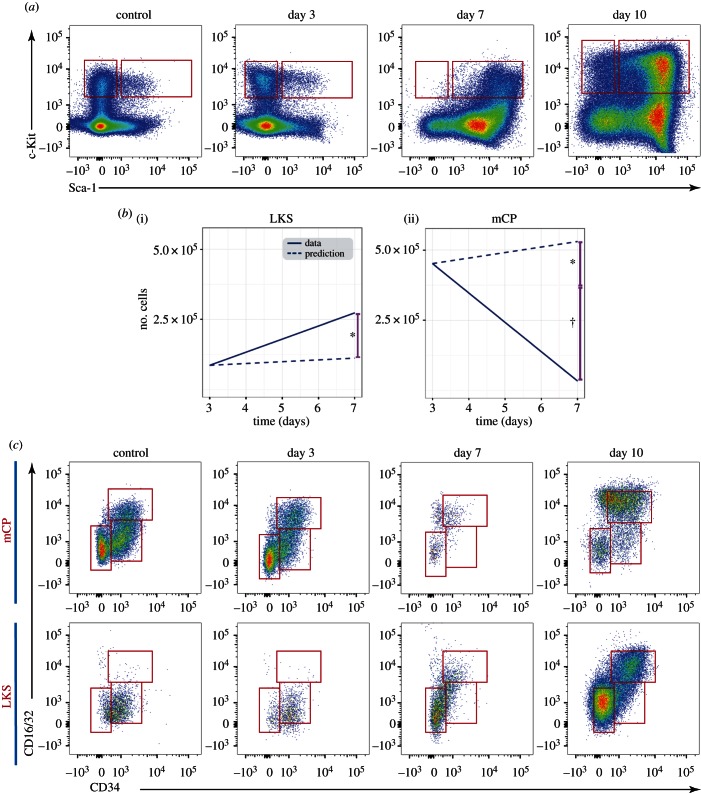
The increased LKS population results from proliferation of HSCs and LMPPs and upregulation of Sca-1 by a proportion of mCPs. (*a*) Representative plots showing the changing pattern in Sca-1 and c-Kit expression in undifferentiated, Lineage^−^ bone marrow haematopoietic cells. Boxes indicate the mCP and LKS gates. (*b*) Predicted (dotted lines) and measured (solid lines) changes in the average number of LKS (i) and mCP (ii) cells from day 3 to day 7 based on the proliferation and apoptosis data and assumptions described in the main text. Asterisks indicate: (i) the amount of LKS cells not accounted for by proliferation and (ii) the same amount of mCP cells that may have upregulated Sca-1 and fallen within the measured LKS population. The cross symbol marks the proportion of mCPs lost from day 3 to day 7. (*c*) Representative plots showing CD16/32 and CD34 in LKS and Lin^−^c-Kit^+^Sca-1^−^ cells during the course of sporozoite *P. berghei* infection. (*a*,*c*) *n* ≥ 30 control, >15 day 3, >20 day 7 and >13 day 10 mice from four independent infections.

Dramatic swelling and parallel loss of phenotypically defined LMPP and mCP populations, respectively, have been reported for other models of infection [8,9] and inflammatory cytokine stimulation [7], and can be qualitatively identified as an overall shift in the proportion of Sca-1^+^ cells within the undifferentiated, c-Kit^+^ lineage^−^ bone marrow cell population (figure 5*a*). However, whether this could be the result of committed, Sca-1^−^ cells re-expressing Sca-1, or whether increased proliferation of the existing LMPP population alone could account for the expanded cell numbers observed, and what the fate of the disappearing cMPs may be remain open questions.

In the case of *P. berghei* infection, the shift towards high levels of Sca-1 expression was most dramatic at day 7 psi, with virtually no Sca-1^−^ cells remaining at this time point. By day 10 psi, however, a wider range of Sca-1 intensities was observed in the Lin^−^ fraction (figure 5*a*). We therefore targeted our examination of population dynamics to the time window between day 3 and day 7, and built a simple predictive model based on the BrdU incorporation rate of LKS and mCPs at these time points to test the hypothesis that proliferation of LMPPs was solely responsible for their increased numbers. For the purpose of this analysis, we considered the LKS population as uniform, because HSCs are a small fraction of it during steady state, and become a much smaller fraction by the time infection has developed (electronic supplementary material, figure S1). We used the LKS proliferation rate observed on day 7 relative to day 3 (electronic supplementary material, figure S2*a*), and made back- calculations based on a series of assumptions to infer the highest possible growth rate for the LKS population: (i) that immediately after sampling at day 3, proliferation jumped to the average elevated levels observed at day 7, (ii) that all BrdU+ cells complete S phase and undergo mitosis within 12 h of sampling, (iii) that no increase in apoptosis took place (consistent with sampling results at day 7; electronic supplementary material, figure S2*b*), and finally (iv) that no LKS cells differentiated into mCP. Even based on these best-case scenario assumptions to model cell growth, the resulting curve did not fit with the observed LKS population size, leaving the majority of LKS cells still unaccounted for on day 7 (figure 5*b*(i)). This analysis indicates that HSC and LMPP proliferation alone is potentially insufficient to drive the expansion of the LMPP/LKS population to the levels we detected.

A possible explanation for the observed excess in the LKS population is overexpression of Sca-1 in the mCP population, with these cells thus ‘masquerading’ as phenotypically defined LKS. To test this hypothesis, we performed a parallel modelling analysis. We assumed that each BrdU+ mCP would complete S phase and mitosis within 12 h of sampling, we applied a constant rate of proliferation based on the average data collected at day 3 (figure 4*b*), and we assumed no overall changes in the rate of differentiation of mCP. The resulting growth curve showed a small increase in mCP numbers, in stark contrast with the data that show a sharp decline in the mCP population size on day 7. Comparison between the LKS and the mCP populations suggested that a relatively small proportion of mCPs upregulating Sca-1 could account for the gap between observed and predicted LKS cells on day 7 (figure 5*b*(ii), asterisk).

We then tested the newly generated hypothesis that the observed LKS cells could indeed be a mixture of genuine LMPPs and ‘masquerading’ mCPs by using flow cytometry to analyse the distribution of mCP markers CD34 and CD16/32 within the LKS population itself (figure 5*c*). During steady state, these two markers clearly label mCP fractions of megakaryocyte/erythrocyte progenitors (MEPs, CD16/32^−^, CD34^−^), common myeloid progenitors (CMPs, CD16/32^intermediate^, CD34^+^) and granulocyte/monocyte progenitors (GMPs CD16/32^bright^, CD34^+^), but this is not the case when the expression of the same markers is analysed in cells within the LKS gate. As we predicted, within this gate already an MEP-like population appears at day 3, and at days 7 and 10 psi the MEP/CMP/GMP pattern of CD16/32 and CD34 expression is clearly recognizable. Interestingly, the few remaining cells in the mCP gate appear to be predominantly GMPs.

These observations raised the question whether not only myeloid but also lymphoid progenitors could change their phenotype during *P. berghei* infection, especially given that CLP-like, c-Kit bright, myeloid-primed progenitors had been previously described following *Plasmodium chabaudi* infection [11]. Indeed, both at days 7 and 10 psi we observed a decrease in IL7Rα^+^ c-Kit^intermediate^ CLPs and we could identify IL7Rα^+^c-Kit^bright^ cells instead (electronic supplementary material, figure S3). Of note, the majority of IL7Rα^+^ cells were Sca-1^+^ (electronic supplementary material, figure S3*a*, bottom row).

### 3.4. Mathematical modelling excludes HSC dynamics alone contribute to the haematopoietic perturbations observed

**Figure 6.**
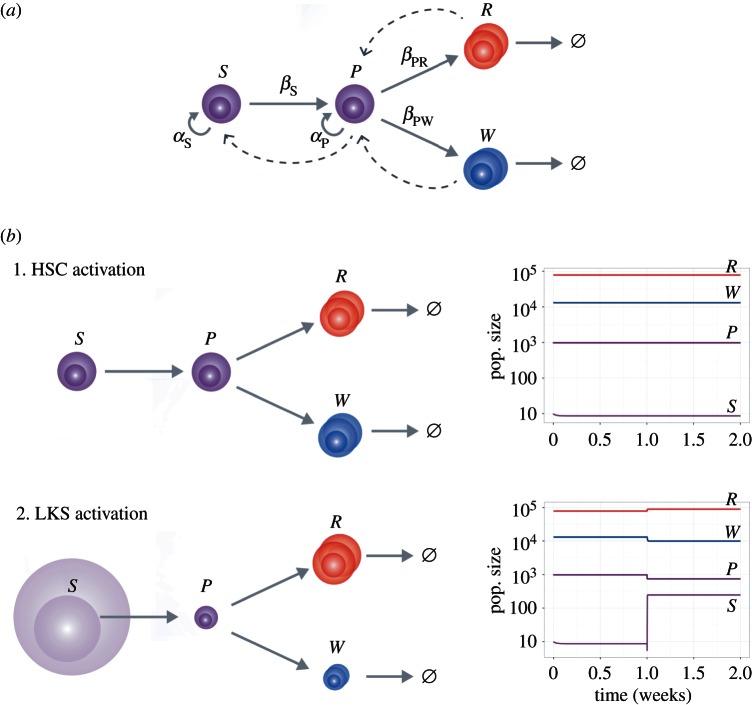
Mathematical model testing the impact of changes in proliferation and differentiation of HSCs alone and HSC + haematopoietic progenitor cell populations combined on downstream haematopoiesis. (*a*) Schematic of the model used, which includes a stem cell population ‘*S*’, an intermediate progenitor population ‘*P*’, and differentiated progeny of red (*R*) and white (*W*) blood cells, and their respective fates (grey arrows). Cells within the *S* and *P* population proliferate at a rate *α*_S_ and *α*_P_ and differentiate *S* into *P* at rate *β*_S_ and *P* into *R* and *W* at rates *β*_PR_ and *β*_PW_. The only fate of *R* and *W* cells is death. Feedback mechanisms from *R* and *W* onto *P* and from *P* onto *S* avoid unbound growth (dotted arrows). (*b*) Diagrammatic representations of the result of altering dynamics of the *S* population alone (1) or of *S* and *P* simultaneously (2). In (1), *α*_S_ and *β*_S_ grow equally, but this has no effect on the overall haematopoietic dynamics. In (2), both *α*_S_ and *β*_S_ and *α*_P_ and *β*_PR_/*β*_PW_ pairs undergo balanced increase, resulting in haematopoietic perturbations.

While it is generally accepted that HSCs are at the origin of all blood cell production, recent work indicates that multipotent progenitors (MPPs, a subpopulation of our LMPPs) sustain the vast majority of haematopoiesis during steady state in adult mice [36,37]. To test whether HSC proliferation alone can account for increased blood cell production and replacement of those differentiated cells lost during the immune response, we generated an ordinary differential equation model of a simplified haematopoietic tree, with population sizes scaled to those of murine haematopoiesis (figure 6). We previously showed that adding multiple intermediate cell populations with intermediate characteristics between HSCs and differentiated cells has little/no effect on the outcome of the model [38]; therefore, and to maintain parsimony, we included only four populations: stem cells (S), progenitor cells (P), and red and white differentiated blood cells (R and W, respectively). Stem and progenitor cells proliferate at their individual rates *α*_S_ and *α*_P_, and differentiate at rates *β*_S_ and *β*_P_. Progenitor cells give rise to both red and white blood cells, with differentiation rates *β*_PR_ and *β*_PW_ (for red and white lineage differentiation, respectively). In steady state, *α*_S_ and *β*_S_ are equal because the HSC population remians constant and, at the population level, self-renewal and differentiation are balanced. The scenario *α* > *β* would lead to increased self-renewal at the population level. Conversely, if *β* > *α*, this would result in a reduction in the HSC population size due to decreased self-renewal. The model also contains two feedback functions, one onto HSCs and one onto progenitors; these decrease the growth rate of each population when the number of cells in the population itself or its direct progeny grows large, thus inhibiting unbounded growth. This is a simple mechanism for population growth control, used widely for dynamical systems [39]. The feedback origin from direct progeny or more distant descendants has little/no effect on the model [39] and therefore, for simplicity, we used feedback from direct progeny.

Within this model, HSC activation is a balanced increase in both proliferation and differentiation rates, which does not affect the size of the HSC population and is consistent with our observations of (i) increase in BrdU+ HSCs (figure 3*c*) and (ii) HSC numbers remaining essentially unchanged (they fluctuated around a constant average—electronic supplementary material, figure S1). One might anticipate that this would be sufficient to lead to a (possibly transient) increase in the downstream populations of progenitor and differentiated cells, as input into the progenitor pool is increased. However, in the model we studied here, when *α*_S_ and *β*_S_ were increased equally (but *α*_P_ and *β*_P_ remained unchanged), this was not observed (figure 6*b*1), suggesting that HSC activation alone cannot explain the dynamics observed *in vivo*.

We next simulated a different scenario, in which not only HSC activation occurs (as before, *α*_S_ and *β*_S_ increase equally), but also proliferation and differentiation of progenitors increase. In this case, we see changes across the haematopoietic hierarchy, including expansion of the HSC population and loss of progenitor cells, as observed *in vivo*. Under these conditions, the model predicted loss of white cells and a modest increase in red blood cells (figure 6*b*2). In this scenario, changes in progenitor dynamics have a more profound effect on the haematopoietic tree than changes exclusively at the HSC level, leading us to propose that progenitor dynamics may be intricately involved in the haematopoietic response to *P. berghei* infection.

### 3.5. Bone marrow and peripheral blood cellularity change in agreement with model predictions

Because our model predicted a loss of differentiated cells, and to gain insight into the possible dynamic changes in differentiated haematopoietic cells, we investigated the overall composition of peripheral blood and bone marrow of *P. berghei*-infected mice.

**Figure 7.**
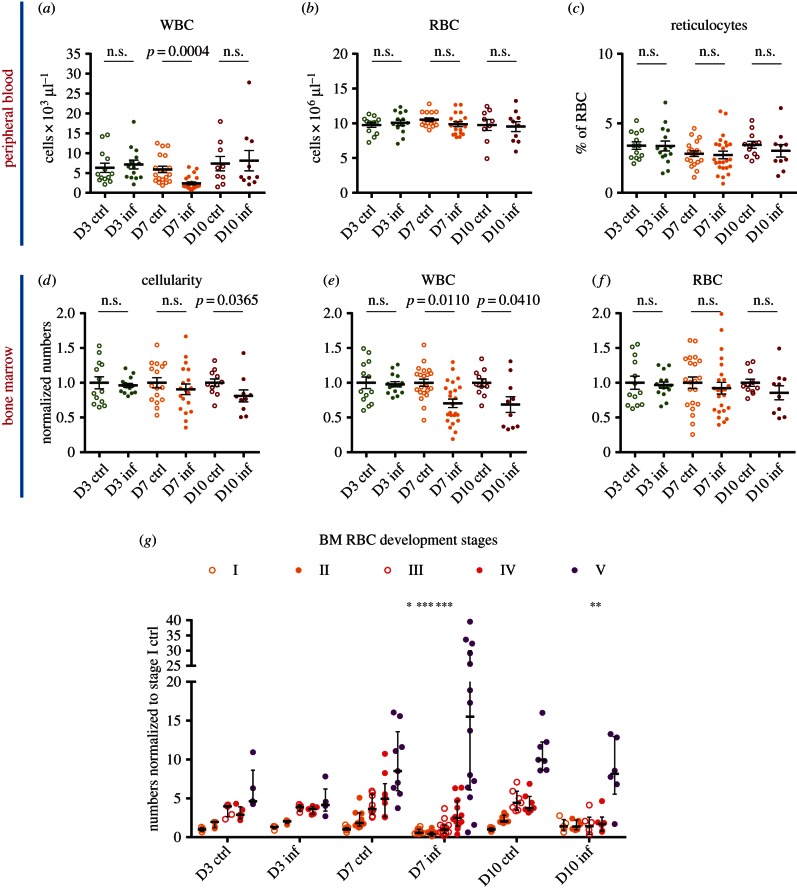
Dynamics of overall bone marrow and peripheral blood populations during sporozoite *P. berghei* infection and onset of a pre-anaemic stage. (*a,b*) Peripheral blood white (*a*) and red (*b*) cell counts in control and infected animals at the indicated days psi. *n* = 13, 19, 9 control and 14, 23, 10 infected mice analysed at days 3, 7 and 10 psi, respectively, in (*a*); 13, 16, 9 control and 14, 18, 10 infected mice analysed at days 3, 7 and 10 psi, respectively, in (*b*). Data pooled from four independent infections. (*c*) Frequency of reticulocytes in peripheral blood, expressed as a percentage of overall red blood cells analysed. *n* = 13, 19, 12 control and 15, 24, 10 infected mice analysed at days 3, 7 and 10 psi, respectively. Data pooled from five independent infections. (*d*) Total bone marrow cellularity of control and infected mice analysed at days 3, 7 and 10 psi. *n* = 13, 16, 11 control and 15, 19 and 10 infected mice. (*e,f*) Bone marrow cell counts for erythrocytes (Ter119+) and stem/progenitor/white overall cell population (Ter119–). *n* = 13, 21, 11 control and 15, 24, 10 infected mice analysed at days 3, 7 and 10 psi, respectively. (*g*) Normalized counts for cells at stages I, II, II, IV and V or erythroid differentiation in the bone marrow of control and infected mice at days 3, 7 and 10 psi. *n* = 5, 9, 7 control and 5, 14, 6 infected mice. Data pooled from three independent infections. *p*-Values for comparison between infected and control values each day are: *p < 0.05; ***p < 0.005, otherwise not significant.

Interestingly, the number of peripheral white blood cells dipped at day 7 psi, in agreement with loss being driven by both insufficient myelopoiesis and immune cell exhaustion/tissue sequestration (figure 7*a*), however the haematocrit values remained constant (figure 7*b*). We next measured the percentage of reticulocytes in peripheral blood, because their increase is a well-known hallmark of *Plasmodium* infection. However, their proportion remained unchanged (figure 7*c*) over the timeframe of this study, in agreement with the overall lack of anaemia.

The overall bone marrow cellularity presented small and statistically non-significant variations, and eventually decreased at day 10 psi (figure 7*d*), due to a notable decrease in overall white blood cells (Ter119−; figure 7*e*). The reduced white cell numbers were accompanied by a reduced CFU-C precursor output, which we observed when we plated bone marrow from control/infected mice at clonal density (not shown). The observed decrease in white blood cells in peripheral blood and especially in the bone marrow was as predicted by the model.

By contrast, and surprisingly for an anaemia-inducing pathogen, but in agreement with model predictions, the overall number of erythroid cells (Ter119+) in the bone marrow remained unchanged in the period of observation (figure 7*f*). This raised the question of how red cell homeostasis could be maintained despite the dramatic loss of committed myeloid progenitors. Initial observation of persistent decline in bone marrow erythroblast precursors, identified through the BFU-E assay (not shown), starting as early as day 3 psi prompted us to investigate bone marrow erythroid differentiation in greater detail.

Despite the number of fully differentiated erythroid cells (stage V based on Ter119, CD71, CD44 expression and cell size) showing little fluctuation, we observed consistent falls in baso-, poly- and ortho-erythroblasts (stages II, III and IV of terminal RBC development [30]) at days 7 and 10 psi (figure 7*g* and electronic supplementary material, figure S4). The progressive and sequential losses in increasingly mature cell populations suggested that the input to the erythroid lineage may have been constrained, in agreement with the observed loss of mCPs, and that if infection were to develop further anaemia would occur. Interestingly, in a transplant setting in which bone marrow from infected animals was injected into lethally irradiated, syngeneic mice, we observed 20% parasitaemia by three weeks post-transplant (i.e. pbi), and severe anaemia and pan-cytopenia (data not shown), indicating that a decline in RBC counts will occur in our experimental model, when the lethal complications of cerebral malaria are avoided.

## 4. Discussion

Infection of a mammalian host causes complex cellular haematopoietic responses, encompassing not only mature immune cells but also their precursors, including stem cells. Malaria infection places additional stress on these populations, causing significant direct and indirect haemolysis of mature erythrocytes. Here we provide a systematic dissection of the murine haematopoietic response to natural infection by *P. berghei* sporozoites, and link responses in the stem and progenitor cell compartments to fluctuations in terminally differentiated white and red blood cells, from the early pre-erythrocytic phase of the disease until a mounting parasitaemia and a pre-anaemic state are detectable.

We found the number of LT-HSCs and LMPPs significantly increased as early as day 3 psi and mCP proliferation at day 2 psi, an indication that perhaps subtle, but critical responses take place in the haematopoietic stem and progenitor cell compartment from very early stages following sporozoite infection and before blood stage parasitaemia is detectable by microscopy. This is consistent with the fact that sporozoite infection of hepatocytes is known to influence immune responses [17,26] and results in both CD8^+^-mediated [40] and a type 1 IFN response producing the antimicrobial cytokine IFNγ as early as 1 day after sporozoite infection [41,42]. Additionally, changes in iron/hepcidin metabolism have been noted prior to RBC infection [43] and they too could contribute to these early responses. Detection of early changes in HSPCs was made possible by our choice of mosquito bite and sporozoites as the means of infection, which importantly is the physiological mechanism of disease transmission. These observations further strongly support the hypothesis that the activation of early haematopoietic stem and progenitor cells during infection is direct and not a late, domino-effect response to the exhaustion of immune cells [3,4].

From the stem cell perspective, perhaps the most interesting observation is that of significant proliferation of primitive HSCs at days 7–10 of infection. Because this proliferation is not accompanied by significant/prolonged expansion, nor death, of cells in this population, our observation is consistent with the hypothesis that the fate of proliferating HSCs could be asymmetric. Whether this results from asymmetry in single cell divisions or at the population level will have to be determined by further studies. Another important question, relevant to hosts that recover from malaria, is whether such balanced but sustained HSC proliferation may have long-term consequences on HSC fitness, given that replication stress has been associated with loss of functionality of aged HSCs [44] and return to quiescence has been associated with HSC functional recovery from interferon exposure [7,34].

Sustained increase in Sca-1^+^ early haematopoietic progenitors and HSC proliferation have been shown to be hallmarks of haematopoietic responses to bacterial and viral infections [7–10,34], and here we report that this is the case also for parasitic infections, specifically *P. berghei*. We show that this is probably the product of the sum of increased proliferation of the LMPP population itself (previously reported only in response to pulsed INFα stimulation [7]), and of re-expression of Sca-1 by some pre-existing mCP cells. Moreover, the qualitative analysis of expression of mCP markers CD16/32 and CD34 in the expanded LMPPs suggests that during *P. berghei* infection the boundary between LMPP and mCP subpopulations is blurred, and we hypothesize that this may result not only from upregulation of Sca-1 by existing mCPs but also from potential accelerated differentiation of LMPPs, such that normal downregulation of Sca-1 is not completed. Myeloid-biased, Sca-1^high^ haematopoietic progenitors were observed during the course of *P. chabaudi* infection [11]. Future studies will reveal whether this is the case for other infections.

The decrease in mCP numbers at day 7 psi, the absence of increased apoptotic proportion, and calculation that only a small proportion of mCPs are likely to upregulate Sca-1 and return to an LMPP-like state, raise the question of how this relatively large cell population may be lost. One possibility is that the cells rapidly differentiate into red and white cells, for which we observed still normal levels in both bone marrow and peripheral blood at day 7 psi, the other is that these cells could have migrated to the spleen. The latter is in agreement with the splenomegaly we recorded from day 7, and with multiple reports of extramedullary haematopoiesis during inflammatory stress [9,19,45,46], including human and rodent *Plasmodium* infections [15,47–51]. Of note, re-appearance of Lin^−^c-Kit^+^Sca-1^−^ cells within the Lin^−^ c-Kit^+^ gate could reflect regeneration of the committed progenitor cell compartment following their initial dramatic perturbation.

We made the unexpected observation that a small proportion of infected mice, which developed parasitaemia within the expected range, did not respond to it by upregulating Sca-1 expression in the bone marrow. Future studies will need to uncover what epigenetic/environmental mechanisms may underpin such variability. We hypothesize that HSPC and immune responses are deeply linked and mice that fail to upregulate Sca-1 also develop aberrant immune responses and may be protected from cerebral complications.

Both our experimental data and mathematical model indicated that multiple points of the haematopoietic cascade are affected simultaneously in response to *P. berghei* sporozoite infection. Some malaria parasites, including *P. berghei*, have been found in bone marrow [52]. This may induce very local ‘cytokine storms’, which may contribute to the multifaceted changes in haematopoietic cell populations that we report, including loss of bone marrow white blood cells and the development of a pre-anaemic state. The latter two observations are in agreement with earlier studies of blood stage *P. berghei* infection showing that bone marrow erythroid colony formation potential decreases rapidly [22].

We conclude that *P. berghei* infection provides an appropriate and biologically relevant model system to further investigate the cellular and molecular mechanisms involved in the complex haematopoietic response to a natural infection.

## Supplementary Material

Supplementary Figures

## References

[1] Wilson A et al. 2008 Hematopoietic stem cells reversibly switch from dormancy to self-renewal during homeostasis and repair. Cell 135, 1118–1129. (doi:10.1016/j.cell.2008.10.048) 19062086 10.1016/j.cell.2008.10.048

[2] Gordon MY, Lewis JL, Marley SB 2002 Of mice and men…and elephants. Blood 100, 4679–4680. (doi:10.1182/blood-2002-08-2517) 12453884 10.1182/blood-2002-08-2517

[3] King KY, Goodell MA 2011 Inflammatory modulation of HSCs: viewing the HSC as a foundation for the immune response. Nat. Rev. Immunol. 11, 685–692. (doi:10.1038/nri3062) 21904387 10.1038/nri3062 PMC4154310

[4] Mirantes C, Passegue E, Pietras EM 2014 Pro-inflammatory cytokines: emerging players regulating HSC function in normal and diseased hematopoiesis. Exp. Cell Res. 329, 248–254. (doi:10.1016/j.yexcr.2014.08.017) 25149680 10.1016/j.yexcr.2014.08.017 PMC4250307

[5] Baldridge MT, King KY, Goodell MA 2011 Inflammatory signals regulate hematopoietic stem cells. Trends Immunol. 32, 57–65. (doi:10.1016/j.it.2010.12.003) 21233016 10.1016/j.it.2010.12.003 PMC3042730

[6] Esplin BL et al. 2011 Chronic exposure to a TLR ligand injures hematopoietic stem cells. J. Immunol. 186, 5367–5375. (doi:10.4049/jimmunol.1003438) 21441445 10.4049/jimmunol.1003438 PMC3086167

[7] Essers MA, Offner S, Blanco-Bose WE, Waibler Z, Kalinke U, Duchosal MA, Trumpp A 2009 IFNalpha activates dormant haematopoietic stem cells *in vivo*. Nature 458, 904–908. (doi:10.1038/nature07815) 19212321 10.1038/nature07815

[8] Rodriguez S et al. 2009 Dysfunctional expansion of hematopoietic stem cells and block of myeloid differentiation in lethal sepsis. Blood 114, 4064–4076. (doi:10.1182/blood-2009-04-214916) 19696201 10.1182/blood-2009-04-214916 PMC2774548

[9] Baldridge MT, King KY, Boles NC, Weksberg DC, Goodell MA 2010 Quiescent haematopoietic stem cells are activated by IFN-γ in response to chronic infection. Nature 465, 793–797. (doi:10.1038/nature09135) 20535209 10.1038/nature09135 PMC2935898

[10] MacNamara KC, Jones M, Martin O, Winslow GM 2011 Transient activation of hematopoietic stem and progenitor cells by IFNγ during acute bacterial infection. PLoS ONE 6, e28669 (doi:10.1371/journal.pone.0028669) 22194881 10.1371/journal.pone.0028669 PMC3237486

[11] Belyaev NN, Brown DE, Diaz A-IG, Rae A, Jarra W, Thompson J, Langhorne J, Potocnik AJ 2010 Induction of an IL7-R + c-Kithi myelolymphoid progenitor critically dependent on IFN-γ signaling during acute malaria. Nat. Immunol. 11, 477–485. (doi:10.1038/ni.1869) 20431620 10.1038/ni.1869

[12] de Bruin AM, Libregts SF, Valkhof M, Boon L, Touw IP, Nolte MA 2012 IFNγ induces monopoiesis and inhibits neutrophil development during inflammation. Blood 119, 1543–1554. (doi:10.1182/blood-2011-07-367706) 22117048 10.1182/blood-2011-07-367706

[13] Maltby S, Hansbro NG, Tay HL, Stewart J, Plank M, Donges B, Rosenberg HF, Foster PS 2014 Production and differentiation of myeloid cells driven by proinflammatory cytokines in response to acute pneumovirus infection in mice. J. Immunol. 193, 4072–4082. (doi:10.4049/jimmunol.1400669) 25200951 10.4049/jimmunol.1400669 PMC4185243

[14] Rashidi NM, Scott MK, Scherf N, Krinner A, Kalchschmidt JS, Gounaris K, Selkirk ME, Roeder I, Lo Celso C 2014 *In vivo* time-lapse imaging of mouse bone marrow reveals differential niche engagement by quiescent and naturally activated hematopoietic stem cells. Blood 124, 79–83. (doi:10.1182/blood-2013-10-534859) 24850759 10.1182/blood-2013-10-534859 PMC4125355

[15] Lamikanra AA, Brown D, Potocnik A, Casals-Pascual C, Langhorne J, Roberts DJ 2007 Malarial anemia: of mice and men. Blood 110, 18–28. (doi:10.1182/blood-2006-09-018069) 17341664 10.1182/blood-2006-09-018069

[16] Grau GE, Behr C 1994 T cells and malaria: is Th1 cell activation a prerequisite for pathology? Res. Immunol. 145, 441–454. (doi:10.1016/S0923-2494(94)80175-4) 7899710 10.1016/s0923-2494(94)80175-4

[17] Langhorne J 1994 The immune response to the blood stages of plasmodium in animal models. Immunol. Lett. 41, 99–102. (doi:10.1016/0165-2478(94)90115-5) 8002055 10.1016/0165-2478(94)90115-5

[18] Stevenson MM 1989 Malaria: host responses to infection. Boca Raton, FL: CRC Press.

[19] Belyaev NN, Biro J, Langhorne J, Potocnik AJ 2013 Extramedullary myelopoiesis in malaria depends on mobilization of myeloid-restricted progenitors by IFN-γ induced chemokines. PLoS Pathog. 9, e1003406 (doi:10.1371/journal.ppat.1003406) 23762028 10.1371/journal.ppat.1003406 PMC3675198

[20] Cromer D, Evans KJ, Schofield L, Davenport MP 2006 Preferential invasion of reticulocytes during late-stage *Plasmodium berghei* infection accounts for reduced circulating reticulocyte levels. Int. J. Parasitol. 36, 1389–1397. (doi:10.1016/j.ijpara.2006.07.009) 16979643 10.1016/j.ijpara.2006.07.009

[21] Harris JV, Bohr TM, Stracener C, Landmesser ME, Torres V, Mbugua A, Moratz C, Stoute JA 2012 Sequential *Plasmodium chabaudi* and *Plasmodium berghei* infections provide a novel model of severe malarial anemia. Infect. Immun. 80, 2997–3007. (doi:10.1128/IAI.06185-11) 22689817 10.1128/IAI.06185-11 PMC3418732

[22] Maggio-Price L, Brookoff D, Weiss L 1985 Changes in hematopoietic stem cells in bone marrow of mice with *Plasmodium berghei* malaria. Blood 66, 1080–1085. 3902119

[23] Playfair JHL 1994 The pathology of malaria: a possible target for immunisation? Immunol. Lett. 43, 83–86. (doi:10.1016/0165-2478(94)00162-6) 7737694 10.1016/0165-2478(94)00162-6

[24] Safeukui I et al. 2015 Malaria induces anemia through CD8^+^ T cell-dependent parasite clearance and erythrocyte removal in the spleen. mBio 6, e02493-14 (doi:10.1128/mBio.02493-14) 25604792 10.1128/mBio.02493-14 PMC4324318

[25] Spence P, Jarra W, Levy P, Nahrendorf W, Langhorne J 2012 Mosquito transmission of the rodent malaria parasite *Plasmodium chabaudi*. Malar. J. 11, 407 (doi:10.1186/1475-2875-11-407) 23217144 10.1186/1475-2875-11-407 PMC3528485

[26] Spence PJ, Jarra W, Levy P, Reid AJ, Chappell L, Brugat T, Sanders M, Berriman M, Langhorne J 2013 Vector transmission regulates immune control of *Plasmodium* virulence. Nature 498, 228–231. (doi:10.1038/nature12231) 23719378 10.1038/nature12231 PMC3784817

[27] Blagborough AM, Churcher TS, Upton LM, Ghani AC, Gething PW, Sinden RE 2013 Transmission-blocking interventions eliminate malaria from laboratory populations. Nat. Commun. 4, 1812 (doi:10.1038/ncomms2840) 23652000 10.1038/ncomms2840 PMC3674233

[28] Sinden RE 1978 The cell biology. In Rodent malaria (eds R Killick-Kendrick, W Peters), pp. 85–168. London, UK: Academic Press.

[29] Hayashi M, Morita T, Kodama Y, Sofuni T, Ishidate M Jr 1990 The micronucleus assay with mouse peripheral blood reticulocytes using acridine orange-coated slides. Mutat. Res. 245, 245–249. (doi:10.1016/0165-7992(90)90153-B) 1702516 10.1016/0165-7992(90)90153-b

[30] Chen K, Liu J, Heck S, Chasis JA, An X, Mohandas N 2009 Resolving the distinct stages in erythroid differentiation based on dynamic changes in membrane protein expression during erythropoiesis. Proc. Natl Acad. Sci. USA 106, 17 413–17 418. (doi:10.1073/pnas.0909296106) 10.1073/pnas.0909296106 PMC2762680 19805084

[31] Grau GE, Lou JN 1994 TNF-Induced microvascular pathology in the TH1-mediated lesions of mouse cerebral malaria—possible new mechanisms. In Cytokines: basic principles and clinical applications. Ares Serono Symposia, Rome, Italy, 1994 (eds S Romagnani, AK Abbas), pp. 265–299. New York, NY: Raven Press.

[32] Yilmaz OH, Kiel MJ, Morrison SJ 2006 SLAM family markers are conserved among hematopoietic stem cells from old and reconstituted mice and markedly increase their purity. Blood 107, 924–930. (doi:10.1182/blood-2005-05-2140) 16219798 10.1182/blood-2005-05-2140 PMC1895895

[33] Oguro H, Ding L, Morrison SJ 2013 SLAM family markers resolve functionally distinct subpopulations of hematopoietic stem cells and multipotent progenitors. Cell Stem Cell 13, 102–116. (doi:10.1016/j.stem.2013.05.014) 23827712 10.1016/j.stem.2013.05.014 PMC3736853

[34] Pietras EM, Lakshminarasimhan R, Techner JM, Fong S, Flach J, Binnewies M, Passegue E 2014 Re-entry into quiescence protects hematopoietic stem cells from the killing effect of chronic exposure to type I interferons. J. Exp. Med. 211, 245–262. (doi:10.1084/jem.20131043) 24493802 10.1084/jem.20131043 PMC3920566

[35] Bryder D, Rossi DJ, Weissman IL 2006 Hematopoietic stem cells: the paradigmatic tissue-specific stem cell. Am. J. Pathol. 169, 338–346. (doi:10.2353/ajpath.2006.060312) 16877336 10.2353/ajpath.2006.060312 PMC1698791

[36] Busch K, Klapproth K, Barile M, Flossdorf M, Holland-Letz T, Schlenner SM, Reth M, Hofer T, Rodewald HR 2015 Fundamental properties of unperturbed haematopoiesis from stem cells *in vivo*. Nature 518, 542–546. (doi:10.1038/nature14242) 25686605 10.1038/nature14242

[37] Sun J, Ramos A, Chapman B, Johnnidis JB, Le L, Ho YJ, Klein A, Hofmann O, Camargo FD 2014 Clonal dynamics of native haematopoiesis. Nature 514, 322–327. (doi:10.1038/nature13824) 25296256 10.1038/nature13824 PMC4408613

[38] MacLean AL, Lo Celso C, Stumpf MP 2013 Population dynamics of normal and leukaemia stem cells in the haematopoietic stem cell niche show distinct regimes where leukaemia will be controlled. J. R. Soc. Interface 10, 20120968 (doi:10.1098/rsif.2012.0968) 23349436 10.1098/rsif.2012.0968 PMC3627104

[39] MacLean AL, Kirk PD, Stumpf MP 2015 Cellular population dynamics control the robustness of the stem cell niche. Biol. Open 4, 1420–1426. (doi:10.1242/bio.013714) 26453624 10.1242/bio.013714 PMC4728354

[40] Overstreet MG, Cockburn IA, Chen YC, Zavala F 2008 Protective CD8T cells against *Plasmodium* liver stages: immunobiology of an ‘unnatural’ immune response. Immunol. Rev. 225, 272–283. (doi:10.1111/j.1600-065X.2008.00671.x) 18837788 10.1111/j.1600-065X.2008.00671.x PMC2597001

[41] Liehl P, Meireles P, Albuquerque IS, Pinkevych M, Baptista F, Mota MM, Davenport MP, Prudencio M 2015 Innate immunity induced by *Plasmodium* liver infection inhibits malaria reinfections. Infect. Immun. 83, 1171–1180. (doi:10.1128/IAI.02796-14) 10.1128/IAI.02796-14 PMC4333462 25583524

[42] Liehl P et al. 2014 Host-cell sensors for *Plasmodium* activate innate immunity against liver-stage infection. Nat. Med. 20, 47–53. (doi:10.1038/nm.3424) 24362933 10.1038/nm.3424 PMC4096771

[43] Portugal S, Drakesmith H, Mota MM 2011 Superinfection in malaria: *Plasmodium* shows its iron will. EMBO Rep. 12, 1233–1242. (doi:10.1038/embor.2011.213) 22081142 10.1038/embor.2011.213 PMC3245699

[44] Flach J et al. 2014 Replication stress is a potent driver of functional decline in ageing haematopoietic stem cells. Nature 512, 198–202. (doi:10.1038/nature13619) 25079315 10.1038/nature13619 PMC4456040

[45] Vos O, Buurman WA, Ploemacher RE 1972 Mobilization of haemopoietic stem cells (CFU) into the peripheral blood of the mouse; effects of endotoxin and other compounds. Cell Tissue Kinet. 5, 467–479. (doi:10.1111/j.1365-2184.1972.tb00385.x) 4569900 10.1111/j.1365-2184.1972.tb00385.x

[46] Jordan S et al. 2013 Natural killer cells are required for extramedullary hematopoiesis following murine cytomegalovirus infection. Cell Host Microbe. 13, 535–545. (doi:10.1016/j.chom.2013.04.007) 23684305 10.1016/j.chom.2013.04.007

[47] Jerusalem CHR 1965 Histo- und biometrische Untersuchungen zur Frage der Autohaemaggression bei Infektionen mit *Plasmodium berghei*. Ann. Soc. belgique de Med. tropicale 45, 405–418. 5855033

[48] Jerusalem CHR, Kretschmar W 1964 Die erythro- und Lymphopoese in der Mausenilz bei *Plasmodium berghei*-infektion. Vorh. Anat. Ges. 59, 95–101. 14247127

[49] Silverman PH, Schooley JC, Mahlmann LJ 1987 Murine malaria decreases hemopoietic stem cells. Blood 69, 408–413. 3801660

[50] Villeval JL, Gearing A, Metcalf D 1990 Changes in hemopoietic and regulator levels in mice during fatal or nonfatal malarial infections. 2. Nonerythroid populations. Exp. Parasitol. 71, 375–385. (doi:10.1016/0014-4894(90)90063-I) 2146142 10.1016/0014-4894(90)90063-i

[51] Villeval JL, Lew A, Metcalf D 1990 Changes in hemopoietic and regulator levels in mice during fatal or nonfatal malarial infections. 1. Erythropoietic populations. Exp. Parasitol. 71, 364–374. (doi:10.1016/0014-4894(90)90062-H) 2146141 10.1016/0014-4894(90)90062-h

[52] Weiss L 1983 Hematopoietic tissue in malaria: facilitation of erythrocytic recycling by bone marrow in *Plasmodium berghei*-infected mice. J. Parasitol. 69, 307–318. (doi:10.2307/3281228) 6343573

