# Supplementary material for: Systematic tracking of altered haematopoiesis during sporozoite-mediated malaria development reveals multiple response points
Source: Open Biol. 2016 Jun 22;6(6):160038. doi: 10.1098/rsob.160038 (PMC4929935; doi:10.1098/rsob.160038)
Supplement: Supplementary Figures [file rsob160038supp1.pdf]

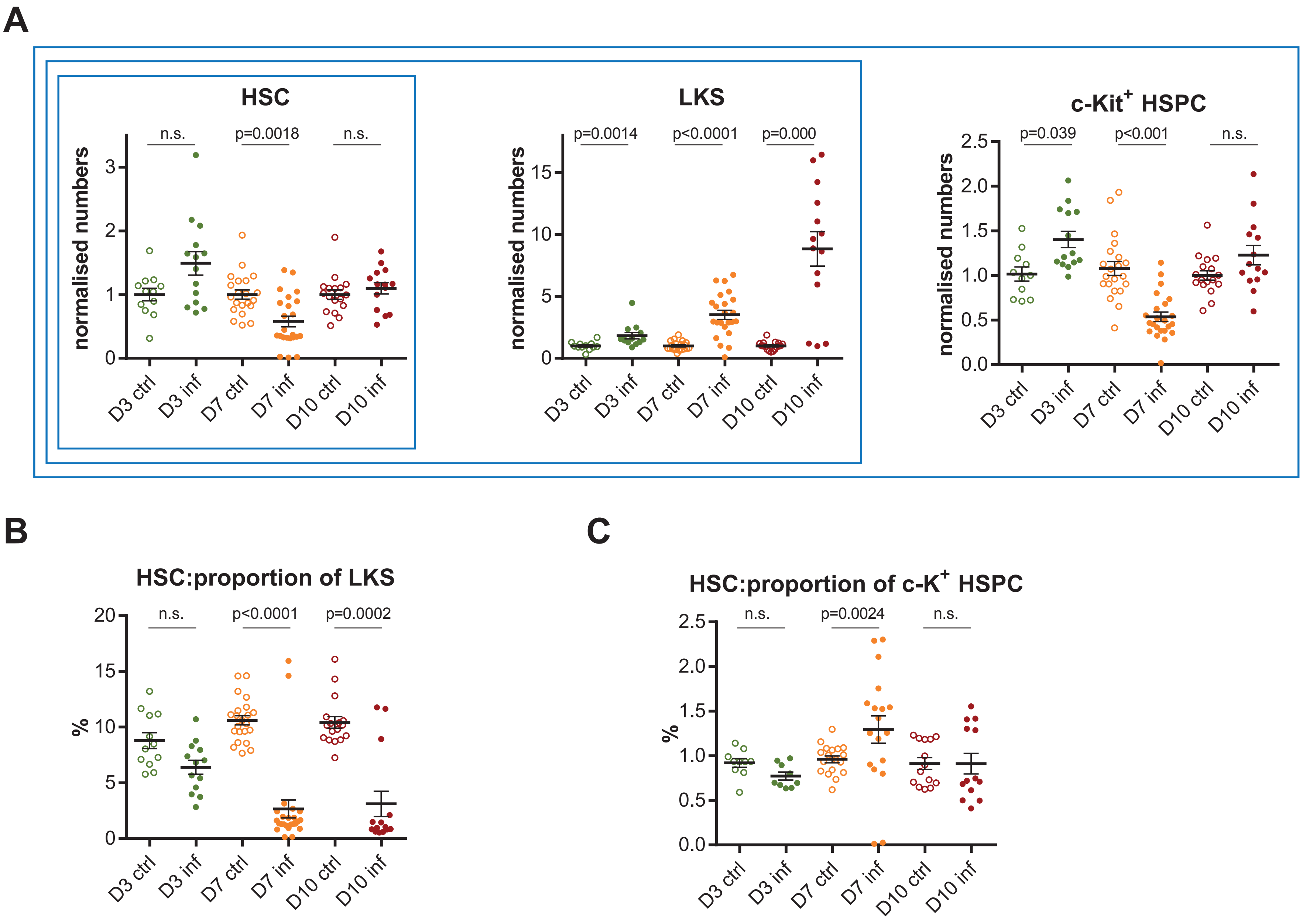

Vainieri et al., Supplementary Figure 1

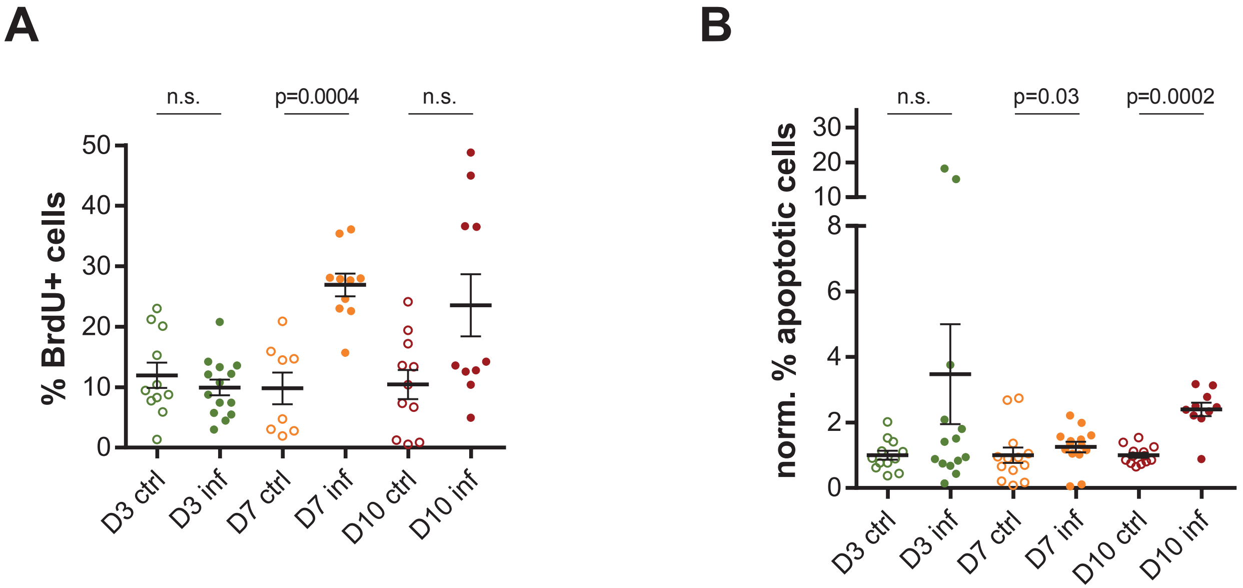

Vainieri et al., Supplementary Figure 2

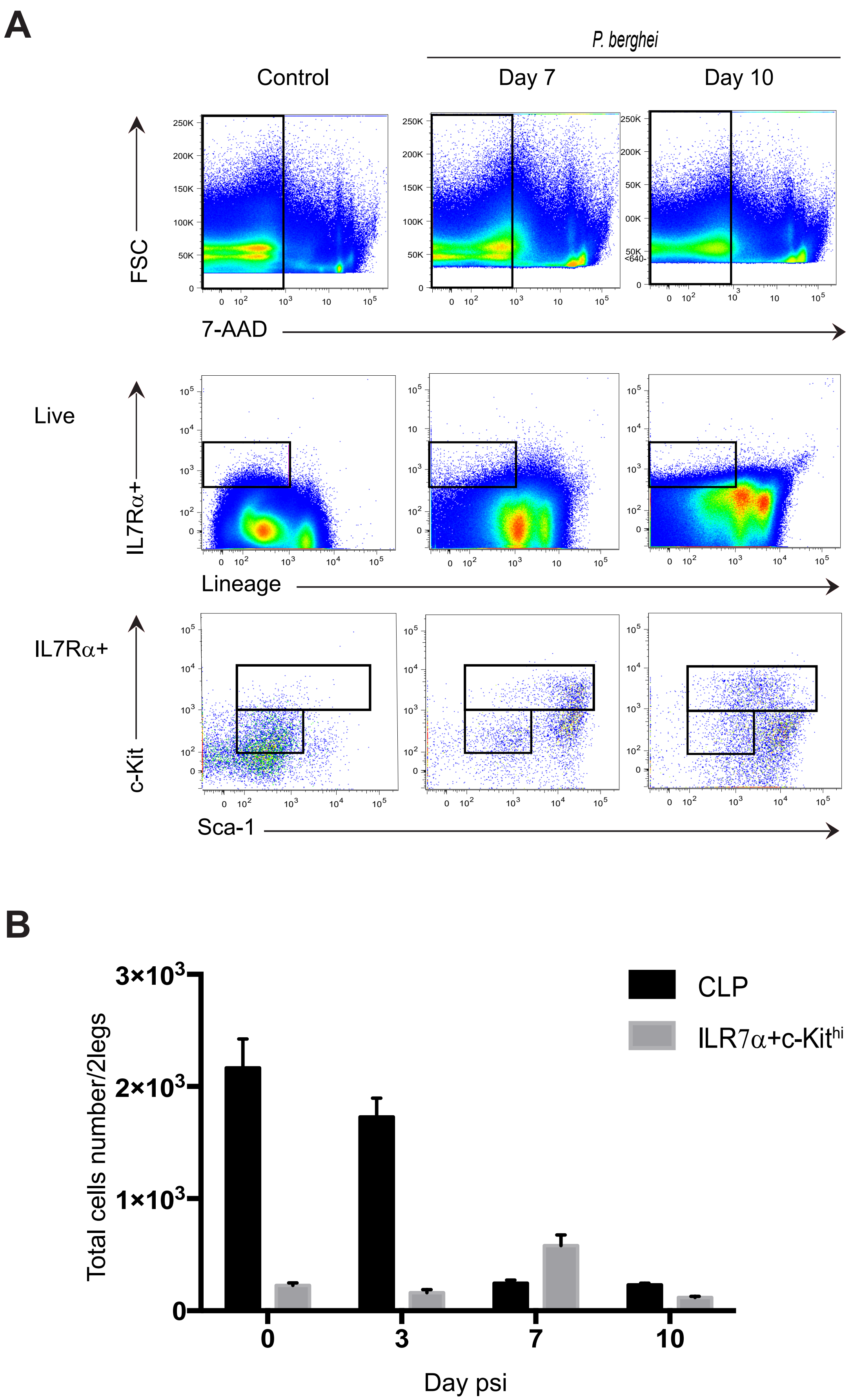

Vainieri et al., Supplementary Figure 3

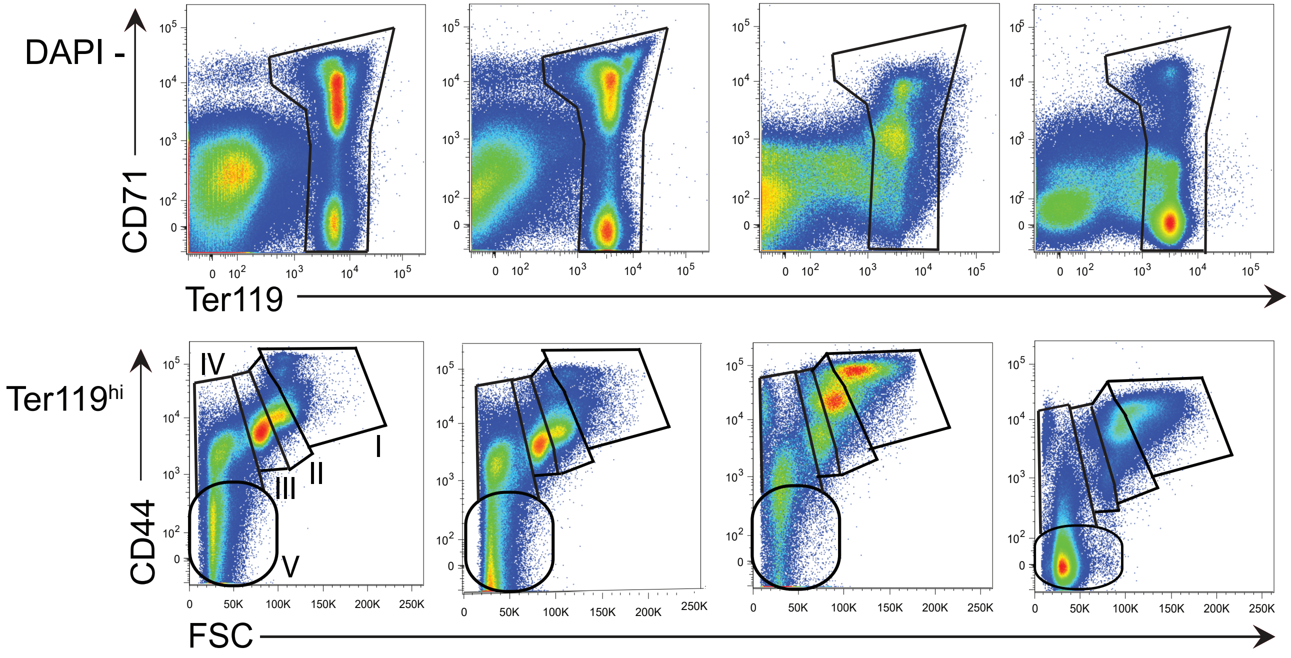

Vainieri et al., Supplementary Figure 4

Supplementary table 1. List of antibodies used for flow cytometry analysis

	Marker	Fluorophore	Clone	Supplier
**HSC**	Lineage-biotin<br>C-Kit<br>Sca1<br>CD150<br>CD48<br>CD34	SA-Pacific Orange<br>APC<br>PercP-Cy5.5<br>BV 605<br>Pacific Blue<br>FITC	N/A<br>2B8<br>D7<br>TC15-12F12.1<br>HM48-1<br>RAM34	eBioscience/L.Tech.<br>BioLegend<br>BioLegend<br>BioLegend<br>BioLegend<br>eBiosciences
**HPC**	Lineage-biotin<br>c-Kit<br>Sca1<br>CD34<br>CD127 (IL7Rα)<br>CD16/32	SA-Pacific Orange<br>APC<br>PercP-Cy5.5<br>FITC<br>APC-eFluor780<br>PE-cy7	N/A<br>2B8<br>D7<br>RAM34<br>A7R34<br>93	eBioscience/L.Tech.<br>BioLegend<br>BioLegend<br>eBioscience<br>eBioscience<br>Biolegend
**HSPC apoptosis**	Lineage-biotin<br>c-Kit<br>Sca1<br>CD150<br>CD48<br>CD34<br>CD127<br>CD16/32<br>AnnexinV<br>7AAD	SA-Pacific Orange<br>APC<br>PercP-Cy5.5<br>BV 605<br>Pacific Blue<br>FITC<br>APC-eFluor780<br>PE-cy7<br>PE	N/A<br>2B8<br>D7<br>TC15-12F12.1<br>HM48-1<br>RAM34<br>A7R34<br>93	eBioscience/L.Tech.<br>BioLegend<br>BioLegend<br>BioLegend<br>BioLegend<br>eBiosciences<br>eBioscience<br>Biolegend<br>BD<br>BD
**RBC development**	CD71<br>CD44<br>Ter119<br>DAPI	FITC<br>PE<br>APC-Cy7	R17217<br>IM7<br>TER-19	Biolegend<br>BioLegend<br>BioLegend<br>Life Technologies
**HSPC proliferation**	Lineage-biotin<br>c-Kit<br>Sca1<br>CD150<br>CD48<br>CD34<br>BrdU	SA-Pacific Orange<br>APC-Cy7<br>PercP-Cy5.5<br>BV 605<br>Pacific Blue<br>FITC<br>APC/Alexa647	N/A<br>2B8<br>D7<br>TC15-12F12.1<br>HM48-1<br>RAM34<br>NA/MoBu1	eBioscience/L.Tech.<br>BioLegend<br>BioLegend<br>BioLegend<br>BioLegend<br>eBiosciences<br>BD/LifeTechnologies
